# Pathology of Free-Living Loggerhead Turtle (*Caretta caretta*) Embryos on the Island of Linosa (Italy)

**DOI:** 10.3390/vetsci12040328

**Published:** 2025-04-02

**Authors:** Frine Eleonora Scaglione, Matteo Cuccato, Erica Longato, Paola Pregel, Daniele Zucca, Stefano Nannarelli, Alessandra De Lucia, Andrea Capobianco Dondona, Marco Pilia, Elisabetta Manuali, Marco Gobbi, Enrico Bollo, Simonetta Appino

**Affiliations:** 1 Frine Eleonora Scaglione, Department of Veterinary Science, University of Torino, Largo P. Braccini 2, 10095 Grugliasco, Italy; matteo.cuccato@unito.it (M.C.); longato.erica@gmail.com (E.L.); paola.pregel@unito.it (P.P.); enrico.bollo@unito.it (E.B.); 2 Institute of Animal Health, University of Las Palmas de Gran Canaria, Calle Perojo 9, 35003 Las Palmas de Gran Canaria, Spain; danielzucca@tiscali.it; 3 Hydrosphera Onlus, Via Appia Nuova 197, 00100 Roma, Italy; stefano.nannarelli@gmail.com (S.N.); alessdl@libero.it (A.D.L.); 4 Istituto Zooprofilattico, Sperimentale del Lazio e, della Toscana “M. Aleandri”, Via Appia Nuova 1411, 00178 Roma, Italy; andrea.capobiancodondona@izslt.it; 5 Farm4trade, Via IV Novembre 33, 66041 Atessa, Italy; 6 Independent Researcher, Via Chiusi 16, 00052 Cerveteri, Italy; marcopiliavet@gmail.com; 7 Istituto Zooprofilattico Sperimentale dell’Umbria e delle Marche, Via G. Salvemini 1, 06126 Perugia, Italy; e.manuali@izsum.it (E.M.); m.gobbi@izsum.it (M.G.); 8 Department of Veterinary Medicine, University of Sassari, Via Vienna 2, 79100 Sassari, Italy; simo@uniss.it

**Keywords:** *Caretta caretta*, embryos, necropsies, renal oxalosis, high temperatures

## Abstract

On the beach of Linosa Island (Italy), 43 loggerhead sea turtle (*Caretta caretta*) unhatched eggs were recovered from nests, formalin-fixed and necropsied. The tissue samples were stained with hematoxylin-eosin (HE), Grocott, von Kossa, periodic acid-Schiff (PAS), and Movat pentachrome stains. Histologically, vacuolar degeneration (100.0%) and increased numbers of melanomacrophages (18.6%) in the liver, and edema (14.0%) in the lungs were observed. Twenty-five kidneys (58.1%) showed deposition of blue amorphous material with HE staining, which also appeared PAS-positive and black with von Kossa staining, allowing a diagnosis of calcium oxalate, confirmed by transmission electron microscopy (TEM). The hepatic lesions may be indicative of toxicosis, infection, or a defense mechanism. A statistically significant association between the nest position and renal oxalosis (renal calcium oxalate deposition) was observed. Renal oxalosis was probably due to the exceptionally high summer temperatures, which were statistically higher compared to the temperatures recorded in the previous two years.

## Simple Summary

The loggerhead turtle (*Caretta caretta*) is a pelagic species found in all temperate oceans, including the Mediterranean Sea. In Italy, the main nesting areas are located on the islands of Linosa and Lampedusa. Despite conservation efforts, the Mediterranean subpopulation of *Caretta caretta* is classified as vulnerable. The main threats to the species are bycatch in fishing, habitat degradation, and climate change. In particular, the rise in environmental temperatures negatively impacts egg incubation and development. This study examined the factors contributing to mortality in unhatched embryos on Pozzolana di Ponente beach, Linosa, with a focus on renal alterations. The results showed signs of liver and kidney damage, likely due to environmental toxins and high temperatures. Global warming is increasing the sand temperature, compromising hatching success and contributing to issues like renal calcium oxalate deposition (renal oxalosys) in embryos. Conservation actions should include protecting the nests and studying the impacts of environmental contaminants to safeguard endangered sea turtles in the Mediterranean.

## 1. Introduction

The loggerhead turtle (*Caretta caretta*) is a pelagic species found in all the oceans of the temperate zone, including the Mediterranean Sea [1]. Several studies have documented the presence of nesting areas in Italy, along the coasts of Puglia, Basilicata, Campania, Calabria, and Sicily [2], but the only consistent nesting sites are the Pozzolana di Ponente beach on Linosa Island and the Spiaggia dei Conigli beach on Lampedusa Island [3].

The International Union for Conservation of Nature (IUCN) identified seven sea turtle species facing endangerment, with a specific focus on three species in the Mediterranean Sea classified as at risk of extinction. In particular, *Caretta caretta* appeared for the first time in 1975 in the category of the IUCN Red List of Italian Vertebrates as critically endangered. After more than three decades of conservation initiatives, the Mediterranean loggerhead turtle sub-population has been designated as vulnerable according to the latest assessment using the IUCN Red List criteria [4]. Several studies have been carried out to ascertain the main causes of death during the life of these species [5], but there is a lack of studies on the earlier stages, such as incubation and hatching. In fact, the loss of laid eggs results in the loss of a significant number of potential future adults already at risk of extinction. Nevertheless, this classification relies on conservation efforts, as substantial threats persist, including fishery bycatch, degradation of marine and terrestrial habitats, climate change, and marine pollution [6]. In comparison with other Mediterranean nesting sites, Italian regions exhibit heightened human activities, including tourism, fishing, and marine traffic, all of which could potentially impact the utilization of coastal habitats by turtles [7,8]. These human activities, combined with natural threats, may jeopardize various stages of sea turtle reproduction, including egg-laying, embryonic development, the occurrence of carapacial abnormalities, and the survival of hatchlings on the beach [5]. For example, during the embryonic stages, the success of hatchlings may be compromised by a range of factors such as human disturbances, with a critical impact on the embryonic development, reducing hatchling success and leading to embryonic mortality [9,10,11]. In recent years, the primary causes described for loggerhead turtles’ mortality are the sea turtle egg fusariosis (STEF) [12,13], organic pollutant contamination [14], and microplastic ingestion [15,16]. In particular, STEF is a recently identified fungal disease associated with mortality in the eggs of endangered sea turtle nests on a global scale [17]. Moreover, the rise in global temperatures as a result of climate change poses a threat to biodiversity worldwide [18]. Non-avian reptiles are more vulnerable to heat stress during incubation due to the absence of parental care and limited behavioral thermoregulation [19]. This has spurred increased research attention to comprehend the consequences of elevated nest temperatures on turtle development. Therefore, the purpose of this work is to evaluate the cause of death of unhatched *Caretta caretta* embryos from Pozzolana di Ponente beach on Linosa Island.

## 2. Materials and Methods

### 2.1. Samples Collection

Forty-three unhatched loggerhead sea turtle eggs (*Caretta caretta*) were found in 2006 on the beach of Pozzolana di Ponente on Linosa Island (35.863101–12.854008) at the end of the summer season. A total of 5 unhatched nests, previously identified and monitored from the moment of oviposition, were part of this study. In particular, the total number of eggs and the depth of eggs in the sand were recorded for each nest.

The nests were manually excavated with a spade and opened and an external visual examination of the eggs was carried out. Subsequently the eggs were carefully opened with the use of scissors for the extraction of the embryos. The samples were then fixed immediately by immersion in 10% buffered formalin (pH 7) to confer stability to the samples and inactivate the enzymes responsible for the autolytic processes. An initial selection was made regarding the specimens collected to exclude subjects clearly in a state of decomposition and therefore unsuitable for subsequent examinations. Fixed samples were sent to the Department of Veterinary Science, University of Turin.

### 2.2. Biometric Data Collection

For each sample, the following biometric data were recorded: standard carapace length (SCL) was measured from the midpoint of the nuchal plate to the caudal portion of the supracaudal scutes of the carapace; standard carapace width (SCW) was obtained by measuring the widest part of the carapace; head length (HL) was measured along the midline, from the posterior tip of the supra-occipital crest to the rostral part of the head (the rhamphotheca of the upper jaw); and head width (HW) was obtained by measuring the widest part of the head. These data were compared with Miller’s tables to evaluate age at death [20].

### 2.3. Gross and Histological Examination

After an external examination of each specimen to search for any lesions or alterations, a necropsy was performed on each turtle embryo following the procedures described by Wyneken [21]. However, the protocol for necropsy was partly modified since the dissected specimens were small in size and still contained some embryonic structures, such as the yolk sac. After gross examination, organs (liver, stomach, intestine, heart, lungs, kidneys, and central nervous system) were paraffin-embedded according to routine histological procedures. Representative sections of each sample were stained with hematoxylin-eosin (HE), Grocott, periodic acid Schiff (PAS), von Kossa, and Movat pentachrome stains. All slides were observed using a Nikon Eclipse E600 light microscope (Nikon Corporation, Tokyo, Japan).

### 2.4. Electron Microscopy

Paraffin-embedded kidney blocks were cut into small pieces and deparaffinized in Sub X (Leica Biosystems, Nussloch, Germany) for 48 h. Samples were retrimmed to pieces no larger than 1 mm, rehydrated in an ethanol series of descending concentrations, and washed in distilled water. Then, they were fixed in 2.5% glutaraldehyde (TAAB) in PBS pH 7.4 for 2 h at 4 °C and post-fixed in 1% osmium tetroxide (OsO_4_) (Next Chimica, Doornhoek, Waterval-Boven, South Africa) in PBS for 2 h at 4 °C. The tissues were dehydrated through ascending grades of ethanol, incubated in propylene oxide (TAAB) for 5 min at room temperature, and embedded in Epon 812. Resin blocks were solidified at 60 °C for 48 h. Semi-thin sections (1 µm) were cut and stained with 1% toluidine blue (*w*/*v*) pH 3.5. Silver-colored ultrathin sections (60–70 nm) were collected onto copper grids coated with a Formvar layer (Electron Microscopy Sciences, Hatfield, PA, USA) and double-stained with uranyl acetate and lead citrate. Samples were examined using and photographed at 80 kV on a CM12 STEM electron microscope (Philips, Eindhoven, The Netherlands).

### 2.5. Temperature Collection

Temperatures on Linosa Island during July and August, when oviposition occurred and when the eggs should have hatched, were monitored and recorded by the weather station on Lampedusa Island from two years before to two years after the egg sampling.

### 2.6. Statistical Analysis

The collected data were analyzed using GraphPad Prism (software version 4.00 for Windows, GraphPad Software, San Diego, CA, USA). Fisher’s test was applied to evaluate the association between the depth at which the eggs were laid and the presence or absence of renal lesions.

The statistical significance of the association between the nests and the presence or absence of renal lesions was evaluated using the chi-square test. The Kruskal–Wallis test (nonparametric ANOVA) and the corresponding Dunn’s post-test were used to evaluate the differences in the distribution of average temperatures in the months of July and August over five consecutive years, using the year of deposition of the examined samples as the reference. A *p* value < 0.05 was considered statistically significant.

## 3. Results

### 3.1. Egg Depth Distributions and Biometric Data

The unhatched eggs were distributed in the sand at different depths. In Nest 1, three eggs were found in the 19–23 cm range, three in the 24–28 cm range, and six in the 29–33 cm range. A total of 12 eggs were recorded. In Nest 2, one egg was found in the 19–23 cm range, two in the 24–28 cm range, three in the 29–33 cm range, and six in the 34–38 cm range. Six eggs were observed in the 39–43 cm range. In total, Nest 2 contained 11 eggs. In Nest 3, one egg was found in the 13–18 cm range, eight in the 24–28 cm range, and three in the 29–33 cm range. One egg was detected in the 34–38 cm range. Nest 3 contained a total of 13 eggs. In Nest 4, three eggs were found in the 24–28 cm range, one in the 29–33 cm range, and two in the 34–38 cm range with a total of six eggs. In the last nest (Nest 5) only one egg was found in the 19–23 cm range (Table 1).

The average temperatures recorded in July and August of 2006 (the year of the study) and in the two preceding and subsequent years were found to differ.

In July, the average temperature two years before the sampling year was 25.7 °C, rising slightly to 25.8 °C the previous year. In the sampling year, the temperature increased significantly to 30.2 °C. The following year, the temperature dropped to 25.7 °C and rose to 26.8 °C two years after.

In August, temperatures were 26.1 °C two years prior and 25.8 °C one year prior to the sampling year. The sampling year recorded a higher temperature of 30.7 °C. In the subsequent years, the temperature rose to 27.2 °C the year after and remained at 27.1 °C two years after (Table 2).

The biometric data showed that 17/43 (39.5%) animals had an SCL between 3.5 and 4.9 cm. These animals were classified as ready to hatch (also referred to as hatchlings) [20], with an approximate range between the 28th and 30th days, corresponding to the very late stage of embryonic development.

All the remaining animals, 26/43 (60.5%), had SCL values between 2.3 cm and 3.5 cm and were accordingly classified as being in the last third of development (in the range between the 22nd and 27th days of development), according to Miller [20].

### 3.2. Gross and Histological Examination

**Figure 1:**
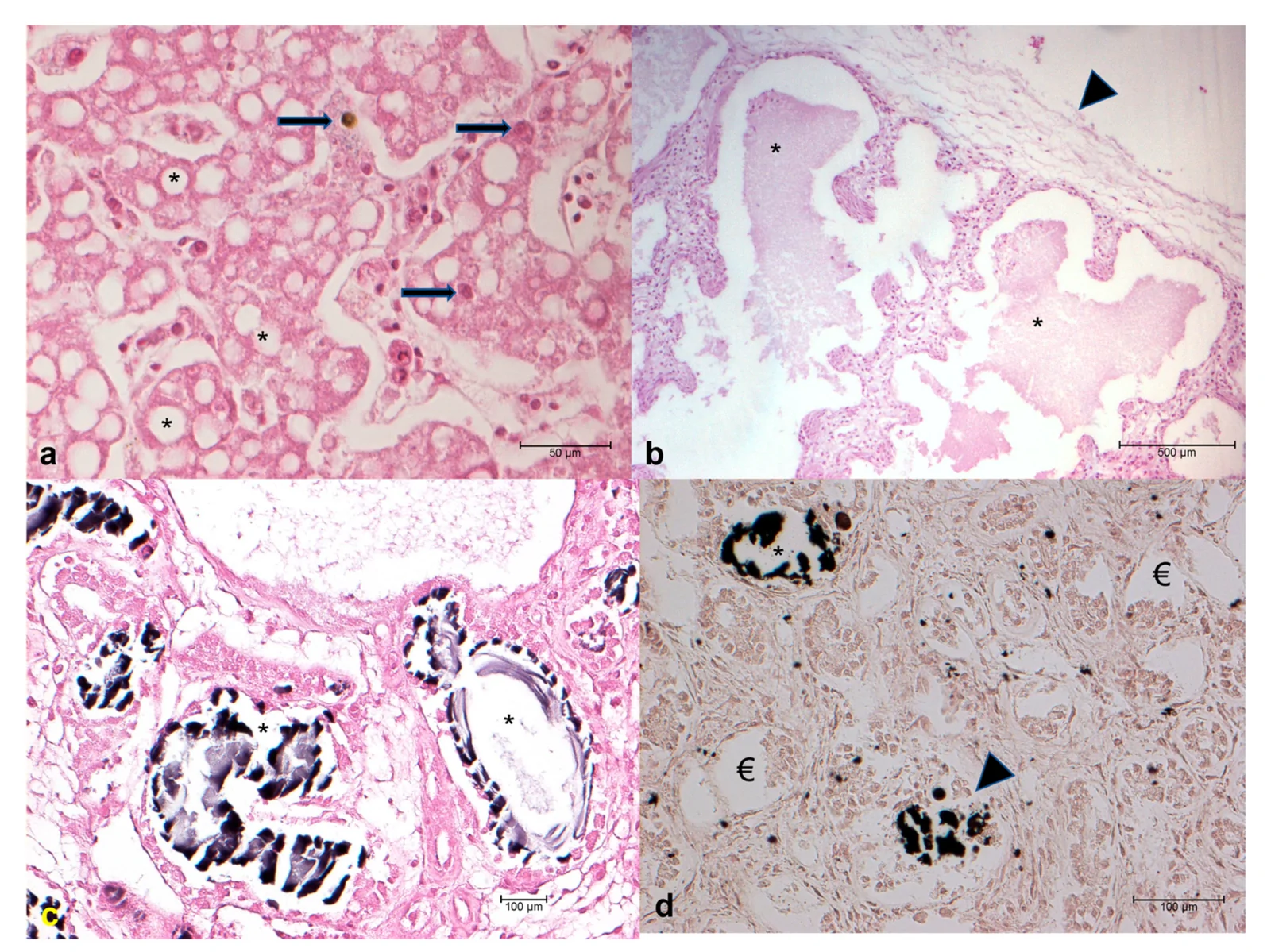
(**a**) Liver melanomacrophages (arrows) and vacuolar degeneration (asterisks) (HE); (**b**) lung oedema (asterisks) (arrowhead: pleural surface) (HE); (**c**) kidney tubular oxalosis (asterisks) (HE); (**d**) kidney glomerular (arrowhead) and tubular oxalosis (asterisks) (€ = normal tubular lumen − von Kossa).

Histological examination of gonads and the central nervous system was not significant due to the heavy degree of autolysis. Stomach and intestine did not show any alterations. In 1/43 (2.3%) cases, a focal non-suppurative infiltration of the heart was observed. An increasing number of melanomacrophages (8/43 cases; 18.6%), hemorrhages (3/43 cases; 7.0%), and vacuolar degeneration (43/43 cases; 100%—Figure 1a) were present in the liver (Figure 1a). Oedema was observed in the lungs of 6/43 (14%) cases (Figure 1b), and 25/43 (58.1%) animals showed glomerular and tubular oxalosis, identified by von Kossa staining as calcium carbonate crystals, involving more than half of the renal parenchyma in 19 out of 25 (76%) animals (Figure 1c,d).

### 3.3. Electron Microscopy

**Figure 2:**
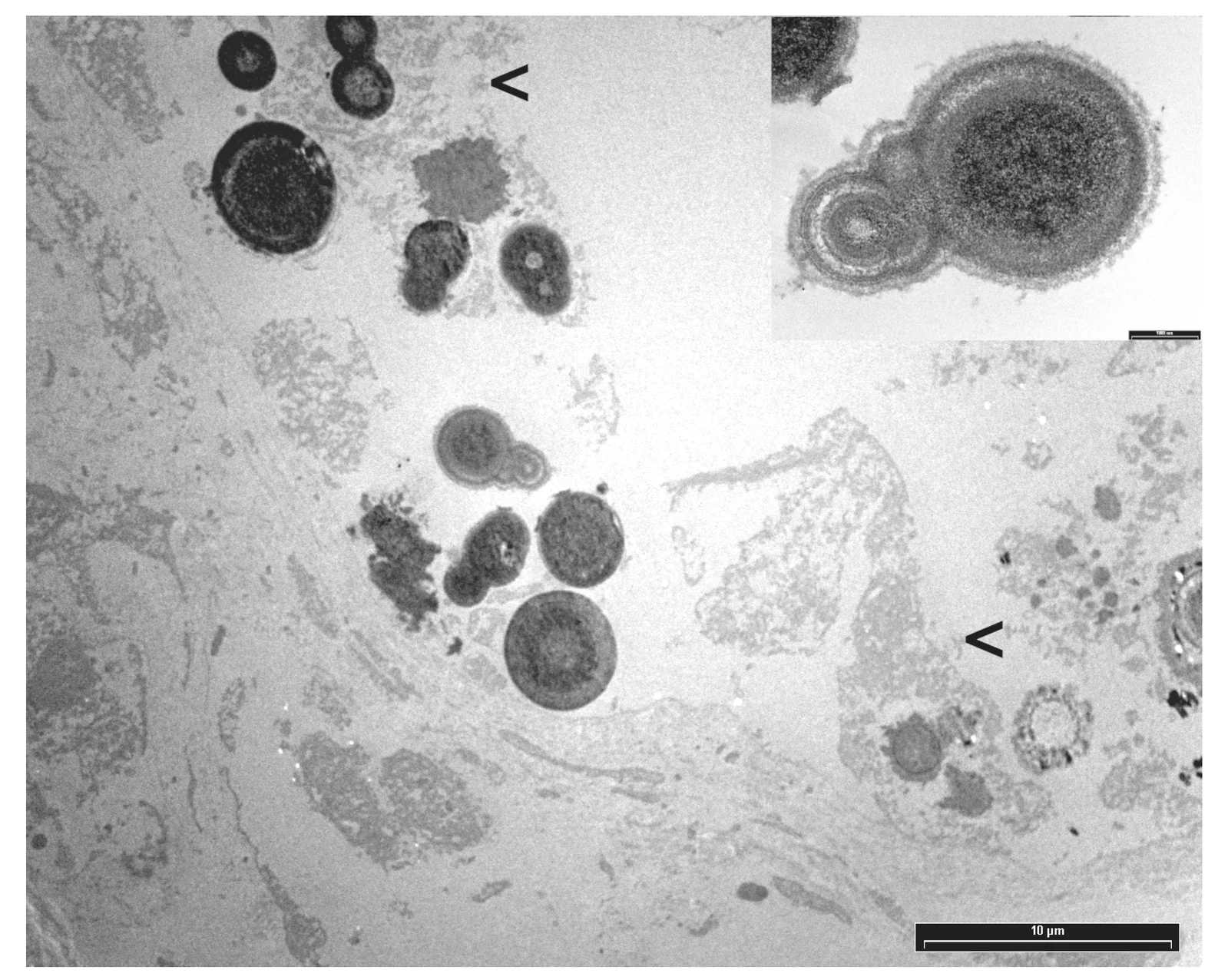
Mineral deposits in the tubular lumen seen as electron dense spherically shaped particles with alternating light and darks rings (inset). Some of them were encased in an organic matrix (arrowheads) (TEM). Tubular epithelial cells undergo degeneration/necrosis by altering the integrity of the cell membrane and releasing amorphous cytoplasmic debris that traps crystals.

Transmission electron microscopy (TEM) revealed mineral deposits characterized by concentric, spherically shaped multilayered rings of crystals with alternating light and dark appearance, often embedded in an organic layer, consistent with calcium oxalate (Figure 2).

### 3.4. Histochemical Staining

Grocott staining did not detect any fungal infection.

### 3.5. Statistical Analysis

Statistical analysis revealed an association between nest position and renal oxalosis (renal calcium oxalate) deposition (*p* < 0.05) and showed significantly higher average temperatures in July and August in the sampling year compared to the other considered years (*p* < 0.001). No statistically significant association was found between nest depth and renal oxalosis when considering eggs laid at shallow depths (13–28 cm) and deep depths (29–43 cm—Table 3).

## 4. Discussion

The most relevant findings in this study were observed in liver and kidney, primarily consisting of vacuolar degeneration of hepatocytes, increasing amount of melanomacrophages and renal oxalosis.

The vacuolar lesions appear to be attributable to a chronic process, since the degeneration appeared widespread and uniform throughout the parenchyma. These types of lesions have been observed for *Caretta caretta* in previous studies and are compatible with a pattern of toxicosis. Merendi and others [22] have observed this degeneration in the liver of *Caretta caretta* stranded on the coast of Emilia Romagna; in that study it was hypothesized that the high concentration of arsenic in the liver, along with traces of other heavy metals, were responsible for the alterations. In addition, Torrent et al. reported severe diffuse vacuolar hepatic degeneration and multifocal necrotizing hepatitis in loggerhead turtles (*Caretta caretta*) with high hepatic arsenic concentrations [23]. The authors suggest that such hepatic damage could have been caused by the inorganic arsenic action, further supporting the link between vacuolar degeneration and chronic toxin exposure. Moreover, Prearo and others [24] observed that high concentrations of these substances were present in liver and kidneys of adult specimens of *Caretta caretta*. Further studies on the presence of persistent organic pollutants have been conducted: Storelli and Marcotrigiano [25] have shown that the concentration of organo-chlorinated pesticides in the organs, particularly liver and kidneys, were higher in juveniles than in adults, while Alam and others [26] described the presence of these substances in eggs of turtles. Environmental pollutants accumulated in females can play an important role during egg development. Furthermore, sea turtles are animals at the top of the food chain, hence these substances can accumulate in the fatty tissue, before being transferred to the egg during its formation as a result of the mobilization of the mother’s energy reserves [27,28]. Moreover, if the sand is further polluted, exposure to contaminants can continue after the deposition, through the gaseous exchanges occurring through the pores of the shell and the shell membranes [29]. Similar findings have been reported in other aquatic species (i.e., mallard ducks and carps) correlating hepatic vacuolar degeneration with chronic toxicosis by selenium or PVC microplastics, respectively [30,31]. In this context, one limitation of this study is the lack of direct contaminant assay data, but previous researchers have documented the correlation between vacuolar degeneration and chronic exposure to toxic substances.

A further interesting result found in this study is the increase of melanomacrophages in 18.6% of liver samples. Factors that may contribute to the increase in number and/or size of these cells are represented by seasonal changes in temperature (as a defense mechanism in adaptive cold-blooded animals) [32], weakness, wasting, stress, chronic inflammation, and chronic diseases caused by bacteria [33]. These data suggest that the increase in melanomacrophages could be due either to a physiological adaptive mechanism against exceptionally hot temperatures during the month of August, when the eggs were about to hatch, or to a defense mechanism against the exposure to toxic substances.

Kidneys showed in 58.1% of cases the deposition in the glomeruli and tubules of amorphous material, identified as crystals of calcium oxalate. It has been shown that in reptiles the most common causes of renal oxalosis include dehydration [34], excessive intake of protein and oxalate, vitamin and calcium deficiency, and bacterial infections [35].

Renal oxalosis is characterized by excess deposition of calcium oxalate crystals within the kidney and the formation of kidney stones (nephrolithiasis) and nephrocalcinosis. Considering that samples were represented by unhatched turtles, their metabolic energy was satisfied by the transfer of nutrients from the mother to the egg [36]. The loggerhead turtle has an omnivorous–carnivorous diet, mainly based on the consumption of shellfishes, molluscs, and at a lesser extent algae [37]. It is therefore unlikely that the renal oxalosis in embryos was due to the vertical transfer of food substances containing calcium oxalate. According to Ackerman [38], the success of incubation for sea turtle eggs depends on the presence of suitable conditions of temperature, humidity, and salinity of the sand. Packard and Packard [39] have demonstrated that a weight loss of more than 40% of the initial weight of the egg (for example due to dehydration), causes a considerable risk for the successful hatching. An analysis of environmental temperatures recorded in the months of July and August (Table 2), in which the eggs were laid, revealed that the average temperature was statistically higher than the previous two and the next two years. Moran and others [40] observed that the critical temperature of the sand beyond which the eggs of *Caretta caretta* do not hatch is 32.4 °C. The sand temperature depends both on the environmental temperature and on its color. Inasmuch as the sand of the Pozzolana di Ponente beach is very dark, being of volcanic origin, a great heat radiation and a high nest temperature are present. The temperature of the sand around the nest also increases in the last stage of embryonic development, following the increases of the metabolic rate of the embryos [41]. Considering that 39.5% of the samples analyzed were hatchlings, while the remaining were in the last stage of incubation, and renal oxalosis can result from dehydration, it can be speculated that the renal alterations were caused by the loss of water from the eggs, due to the abnormally high environmental temperatures. While the study finds no significant association between egg depth and renal oxalosis, Table 3 indicates a high prevalence of oxalosis in deeper eggs (e.g., 6/6 at 39–43 cm). This observed trend suggests that deeper nests may experience distinct microclimatic conditions, which could potentially influence the development of oxalosis, warranting further investigation.

The absence of microbiological or molecular analyses in this study limits the ability to establish a causal link between the observed lesions and environmental factors. This choice was necessitated by sampling difficulties in the field (lack of electricity) and the condition of the samples (the eggs were only removed after it was certain that they would no longer hatch to avoid the risk of harming embryos still capable of hatching). Despite these limitations, the study provides valuable insights into the observed lesions and contributes to the understanding of potential environmental factors affecting the species, highlighting the need for further research with improved methodologies.

## 5. Conclusions

So far, discussions on the effects of global warming on sea turtle populations have primarily focused on the loss of egg-laying sites due to rising sea levels and tides, as well as alterations in sex ratios caused by the resulting increase in sand and nest temperatures [42]. In summary, climate change and human-induced impacts are considered among the most significant threats to the well-being of sea turtles, and should therefore be prioritized in research and conservation management efforts. Furthermore, identifying potential pathogens that pose a threat to endangered sea turtle species, influenced by both global warming and human activities, is essential in developing effective conservation strategies. The findings of this study may indicate a potential threat to loggerhead sea turtle nesting areas in the Mediterranean basin, a phenomenon likely linked to global warming.

Given the endangered status of the loggerhead sea turtle and Linosa’s importance as a key nesting site, further studies are needed to determine the critical temperature for successful hatching. Moreover, research on sand pollution and its toxicity to turtles is required to identify necessary corrective actions for their protection in the Mediterranean.

## Figures and Tables

**Table 1:** Number and depth of the eggs for each nest.

Depth	Nest 1	Nest 2	Nest 3	Nest 4	Nest 5
13–18 cm	-	-	1	-	-
19–23 cm	3	-	-	-	1
24–28 cm	3	-	8	3	-
29–33 cm	6	2	3	1	-
34–38 cm	-	3	1	2	-
39–43 cm	-	6	-	-	-
Total	12	11	13	6	1

**Table 2:** Average monthly temperatures in the months of July and August on the island of Linosa.

JULY			AUGUST		
**Year**	Average Temperature	SD	Year	Average Temperature	D
2 years before	25.7 °C	1.15	2 years before	26.1 °C	0.94
1 year before	25.8 °C	1.18	1 year before	25.8 °C	1.07
Sampling year	30.2 °C	0.73	Sampling year	30.7 °C	1.28
1 year after	25.7 °C	1.64	1 year after	27.2 °C	1.51
2 years after	26.8 °C	1.15	2 years after	27.1 °C	0.76

**Table 3:** Number and depth of the eggs testing positive for renal oxalosis in each nest.

Depth	Nest 1	Nest 2	Nest 3	Nest 4	Nest 5
13–18 cm	-	-	1 (out of 1)	-	-
19–23 cm	2 (out of 3)	-	-	-	0 (out of 1)
24–28 cm	3 (out of 3)	-	3 (out of 8)	1 (out of 3)	-
29–33 cm	3 (out of 6)	2 (out of 2)	1 (out of 3)	0 (out of 1)	-
34–38 cm	-	2 (out of 3)	0 (out of 1)	1 (out of 2)	-
39–43 cm	-	6 (out of 6)	-	-	-
Total	8 (out of 12)	10 (out of 11)	5 (out of 13)	2 (out of 6)	0 (out of 1)

## Data Availability

The datasets presented in this study can be found in online repositories, accessible on request.

## Abbreviations

The following abbreviations are used in this manuscript:

IUCN	International Union for Conservation of Nature
SCL	standard carapace length
SCW	standard carapace width
HL	head length
HW	head width
HE	hematoxylin-eosin stain
PAS	periodic acid Schiff
TEM	transmission electron microscopy
