# Correction: Scaglione et al. Pathology of Free-Living Loggerhead Turtle (*Caretta caretta*) Embryos on the Island of Linosa (Italy). *Vet. Sci.* 2025, *12*, 328

**DOI:** 10.3390/vetsci13101055

**Published:** 2026-10-08

**Authors:** Frine Eleonora Scaglione, Matteo Cuccato, Erica Longato, Paola Pregel, Daniele Zucca, Stefano Nannarelli, Alessandra De Lucia, Andrea Capobianco Dondona, Marco Pilia, Elisabetta Manuali, Marco Gobbi, Enrico Bollo, Simonetta Appino

**Affiliations:** 1 Frine Eleonora Scaglione, Department of Veterinary Science, University of Torino, Largo P. Braccini 2, 10095 Grugliasco, Italy; matteo.cuccato@unito.it (M.C.); longato.erica@gmail.com (E.L.); paola.pregel@unito.it (P.P.); enrico.bollo@unito.it (E.B.); 2 Institute of Animal Health, University of Las Palmas de Gran Canaria, Calle Perojo 9, 35003 Las Palmas de Gran Canaria, Spain; danielzucca@tiscali.it; 3 Hydrosphera Onlus, Via Appia Nuova 197, 00100 Roma, Italy; stefano.nannarelli@gmail.com (S.N.);; 4 Istituto Zooprofilattico, Sperimentale del Lazio e, della Toscana “M. Aleandri”, Via Appia Nuova 1411, 00178 Roma, Italy; andrea.capobiancodondona@izslt.it; 5 Farm4trade, Via IV Novembre 33, 66041 Atessa, Italy; 6 Independent Researcher, Via Chiusi 16, 00052 Cerveteri, Italy; marcopiliavet@gmail.com; 7 Istituto Zooprofilattico Sperimentale dell’Umbria e delle Marche, Via G. Salvemini 1, 06126 Perugia, Italy; e.manuali@izsum.it (E.M.); m.gobbi@izsum.it (M.G.); 8 Department of Veterinary Medicine, University of Sassari, Via Vienna 2, 79100 Sassari, Italy; simo@uniss.it

## Reason for Correction

The authorship list of the published article has been corrected to include Dr. Capobianco Dondona, who was inadvertently omitted. The article is based on field activities conducted in 2008 on juvenile sea turtles in Linosa, during which Dr. Capobianco Dondona, then the veterinarian responsible for the local Sea Turtle Rescue Centre, conceived and planned the sampling of post-mortem material, contributed to the design of the sampling and preservation methodology, coordinated the field activity, and organised the collection and shipment of the samples to the University of Turin, where the analyses were performed. The manuscript [1] was prepared and submitted approximately seventeen years later, without the original field team being contacted. Consequently, Dr. Capobianco Dondona was not informed of the submission and had no opportunity to review the manuscript, verify the author list, or approve the final version before publication. The omission was an unintentional oversight and was acknowledged by the corresponding author, who requested the correction. The corrected authorship list, affiliations, and author contribution statement are provided below. The scientific content of the article remains unchanged.
